# Supplementary material for: Change of Direction Speed and Technique Modification Training Improves 180° Turning Performance, Kinetics, and Kinematics
Source: Sports (Basel). 2021 May 24;9(6):73. doi: 10.3390/sports9060073 (PMC8225134; doi:10.3390/sports9060073)
Supplement: Supplementary file 1 [file sports-09-00073-s001.zip › Supplementary material 1 - Training intervention.pdf]

Supplementary material 1. COD speed and technique modification 6-week training intervention

Week	COD Emphasis	Drills	Intensity<br><br>(perceived speed)	Total Distance (m)	Number of Decelerations and CODs
**Week 1**	• Drills specific to deceleration phase, before adding turn and reacceleration<br>• Submaximal/ pre-planned emphasising key aspects of technique<br>• Progressive increase in COD angle and approach velocity	1. 10 × 5-m acceleration to deceleration (2 reps a go)<br>2. 12 × 5-m acceleration to side-steps (20-60˚) – 5-m exit to deceleration – 2 reps at 20°, 40°, 60˚<br>3. 8 × 5-m acceleration to 135° turns – 5-m exit to deceleration<br>4. 2 × Max effort 135° pivot each leg, max effort 60° cut each leg	50-75%	290	29 and 24
**Week 2**		1. 4 × 5-m acceleration to deceleration<br>2. 4 × 10-m acceleration to deceleration<br>3. 12 × 5-m acceleration to side-steps (45°, 60°, and 80˚) – 5-m exit to deceleration<br>4. 8 × 5-m acceleration to 135° turns – 5-m exit to deceleration<br>5. 2 × Combination: 5-m 60° cut, to 180° turn, to 60° cut, to Dec	75%+	300	30 and 26
**Week 3**	• Pre-planned drill performed maximally<br>• Introduction of unanticipated generic stimuli (auditory or visual)<br>• Unanticipated performed submaximally	1. 4 × 5- and 10-m acceleration to deceleration<br>2. 4 × 2.5-10-m acceleration to deceleration- react to coach shout<br>3. 16 × 5-m acceleration to side-steps (45° and 90˚) – 5-m exit to deceleration<br>4. 4 × 5-m combined 180˚ - 2 x 180° per rep<br>5. 3 × 20-m tunnel drill – side-stepping past 4 opponents every 5-m	75-100%	320-350	28 and 36
**Week 4**		1. 4 × 5-15 m unanticipated decelerations – partner stimuli*<br>2. 3 × zig-zag runs (two 45˚ cuts) – 5-m exit to deceleration (15-m total) – one partner chase<br>3. 3 × zig-zag runs (two 90˚ cuts) – 5-m exit to deceleration (15 m total) – one partner chase<br>4. 6 × unanticipated 70° cuts (coach body position) – 5-m entry and exit<br>5. 3 × pro-agility (5-m approach to 180°, 5-m reaccelerate to 180°, to 10-m deceleration)	100%	260-300	19 and 24
**Week 5**	• Unanticipated drills performed maximally<br>• Introduction of sport specific stimuli – opponent	1. 4 × 5-15 m unanticipated decelerations – partner stimuli*<br>2. 3 × zig-zag runs (two 45˚ cuts) – 5-m exit to deceleration (15-m total) – one partner chase<br>3. 3 × zig-zag runs (two 90˚ cuts) – 5-m exit to deceleration (15-m total) – one partner chase<br>4. 4 × unanticipated 70° cuts (coach body position) – 5-m entry and exit<br>5. 4 × unanticipated 70° cuts (follow opponent) – 5-m entry and exit*<br>6. 2 × 180° races – two turns every 5-m to 5-m deceleration	100%	260-300	26 and 28
**Week 6**		1. 4 x 5-15-m unanticipated decelerations – partner stimuli- two decelerations per rep*<br>2. 8 x 5-m acceleration to side steps (45˚) – 5-m exit to deceleration<br>3. 4 × Modified L runs – anticipated - (5-m acceleration to 90˚ cut, 5-m acceleration to 180˚ turn – 5-m acceleration to 90˚ cut – to 5-m deceleration<br>4. 6 × X-drill – two cuts/turns in a square – partner follow*	100%	270-330	28 and 36

		5. 2 × 180° races – two turns at any point across 10-m distance – partner lead*			

Additional information:

1. 30-60 seconds' rest provided between 100% effort reps. 2 minutes' rest provide between exercises
2. All CODs and decelerations to be performed with the aim of modified braking and COD strategy
3. Feedback to be provided to each player after each rep regarding braking strategy/ COD technique

Key: * = Alternate between leading and reacting / attacking and defending; COD = Change of direction; PFC = Penultimate foot contact; Dec: Deceleration

Supplementary material 1b. Jump-landing warm-up drills performed during COD speed and technique modification training intervention

Week	Warm-up jump-landing plyometric drills
**Week 1**	Jump-landing: 10-m of:<br><br>1. Bilateral broad jump<br>2. Bilateral broad jump – zig-zag<br>3. Bilateral broad jump - lateral<br>4. Single-leg hop and hold – forwards<br>5. Single-leg hop and hold – zig-zag<br>6. Single-leg cutting push off action and hold
**Week 2**	Jump-landing: 10-m of:<br><br>1. Bilateral broad jump<br>2. Bilateral broad jump – zig-zag<br>3. Bilateral broad jump - lateral<br>4. Single-leg hop and hold – forwards<br>5. Single-leg hop and hold – zig-zag<br>6. Single-leg cutting push off action and hold
**Week 3**	Jump-landing: 12-m of:<br><br>1. Bilateral broad jump (reactive in pairs)<br>2. Bilateral broad jump – zig-zag<br>3. Bilateral broad jump - lateral<br>4. Single-leg hop and hold – forwards<br>5. Single-leg hop and hold – zig-zag<br>6. Single-leg cutting push off action and hold
**Week 4**	Jump-landing: 12-m of:<br><br>1. Bilateral broad jump (reactive in pairs)<br>2. Forward tuck-jumps<br>3. Zig-zag tuck-jumps<br>4. Single-leg hop and hold – forwards<br>5. Single-leg hop and hold – zig-zag<br>6. Single-leg cutting push off action continuous
**Week 5**	Jump-landing: 12-m of:<br><br>1. Bilateral broad jump (reactive in pairs)<br>2. Forward tuck-jumps<br>3. Zig-zag tuck-jumps<br>4. Single-leg hop and hold – forwards – double hop<br>5. Single-leg hop and hold – zig-zag<br>6. Single-leg cutting push off action continuous<br>7. Crossover hop and hold
**Week 6**	Jump-landing: 12-m of:<br><br>1. Bilateral broad jump (reactive in pairs)<br>2. Forward tuck-jumps<br>3. Zig-zag tuck-jumps<br>4. Single-leg hop and hold – forwards – double hop<br>5. Single-leg hop and hold – zig-zag<br>6. Single-leg cutting push off action continuous<br>7. Crossover hop and hold
