# Supplementary material for: Change of Direction Speed and Technique Modification Training Improves 180° Turning Performance, Kinetics, and Kinematics
Source: Sports (Basel). 2021 May 24;9(6):73. doi: 10.3390/sports9060073 (PMC8225134; doi:10.3390/sports9060073)
Supplement: Supplementary file 1 [file sports-09-00073-s001.zip › Supplementary material 2 - Methods and variables.pdf]

Supplementary material 2. Definitions and calculations for kinetic and kinematic variables examined during turning

	Variable	Foot contact	Abbreviation	Definition or calculation
Sagittal plane joint angles	Knee flexion angle; hip flexion angle	FFC/PFC	KFA, HFA	Derived from the following order of rotations: flexion (+)/extension (-). Absolute angle of two segments relative to a vertical line. Greater values indicate increased hip flexion, knee flexion.
GRF	Mean horizontal braking force (Fx)	PFC	Mean HBF	Average normalised HGRF (Fx) during weight acceptance
	Mean horizontal propulsive force (Fx)	FFC	Mean HPF	Average normalised HGRF (Fx) during push-off
	Horizontal to vertical braking/propulsive force ratio	PFC and FFC	-	Horizontal force / Vertical force (greater value = greater horizontal force contribution)
	Ground contact time	PFC and FFC	GCT	Duration from IC to toe-off
Trunk, pelvis, and foot variables	Lateral trunk flexion	FFC	-	Angle of trunk relative to vertical line perpendicular to the pelvis in frontal plane: (0˚) upright / (+) medial trunk flexion away from plant foot/ (-) lateral trunk flexion towards plant foot. At IC.
	Forward trunk inclination angle	FFC	-	Angle of trunk relative to a vertical line, (+) forward trunk lean/ (-) backward trunk lean
	Pelvic rotation	FFC	-	Angle of pelvis in transverse plane relative to global coordinate system. (0˚) straight and perpendicular, (+) rotation towards intended direction of travel (-) rotation away from intended direction of travel. At IC.
	Lateral foot plant distance	FFC	-	Lateral distance from initial foot contact of foot COM to proximal end of pelvis
	Initial foot progression angle	FFC	IFPA	Angle of foot progression relative to global coordinate system: straight (0°)/inward rotation (+)/outward rotation (-) angle (°). At IC.
Velocity/COM	Horizontal velocity of COM	PFC and FFC	-	The first derivative of the model COM (combined lower-limb and trunk model) position was computed to derive anterior-posterior (x), vertical (z), and ML (y) over the PFC and FFC. Resultant horizontal plane velocity was calculated using the following formula: $\sqrt{((\text{COM vel }(x)^2) + (\text{COM vel }(y)^2))}$ to provide a "velocity profile" along the path of the participants COM during the cut. Model COM velocity at PFC touch-down (approach), FFC touch-down, and toe-off in FFC (exit).

Key: PFC: Penultimate foot contact; FFC: Final foot contact; COM: Centre of mass; COD: Change of direction; IC: Initial contact; GRF: Ground reaction force; HGRF: Horizontal GRF; MLGRF: Medio-lateral GRF; vel: velocity; ROM: Range of motion.
